# Causal association of sleep traits with the risk of thyroid cancer: A mendelian randomization study

**DOI:** 10.1186/s12885-024-12376-6

**Published:** 2024-05-17

**Authors:** Liang Zong, Guiping Liu, Hongsheng He, Deliang Huang

**Affiliations:** 1 College of Otolaryngology Head and Neck Surgery, Chinese PLA General Hospital, 28 Fuxing Road, Beijing, 100853 China; 2 National Clinical Research Center for Otolaryngologic Diseases, Beijing, 100853 China; 3 Zhantansi Outpatient, Central Medical District of Chinese, PLA General Hospital, Beijing, 100832 China; 4 Zhejiang Shaoxing Topgen Biomedical Technology Co., Ltd, Shanghai, 201321 China

**Keywords:** Sleep traits, Thyroid cancer, Mendelian randomization, Single nucleotide polymorphism

## Abstract

**Background:**

This study was to explore the causal associations of sleep traits including sleep duration, snoring, chronotype, sleep disorders, getting up in the morning, sleeplessness/insomnia and nap during day with the risk of thyroid cancer based on Mendelian randomization (MR) analysis.

**Method:**

Summary single nucleotide polymorphism (SNP)-phenotype association data were obtained from published genome-wide association studies (GWASs) using the FinnGen and UK Biobank databases. A series of screening processes were performed to select qualified SNPs strongly related to exposure. We applied the inverse variance weighted (IVW), the Mendelian Randomization robust adjusted profile score (MR-RAPS), the Mendelian randomization pleiotropy residual sum and outlier (MR-PRESSO), and the Weighted Median to estimate the causal links between sleep traits and the risk of thyroid cancer. Odds ratio (OR) and 95% confidence interval (CI) were calculated.

**Results:**

The IVW results showed that getting up in the morning (OR = 0.055, 95%CI: 0.004–0.741) and napping during day (OR = 0.031, 95%CI: 0.002–0.462) were associated with decreased risk of thyroid cancer in the Italian population. A 1.30-h decrease of sleep duration was associated with 7.307-fold of thyroid cancer risk in the Finnish population (OR = 7.307, 95%CI: 1.642–32.519). Cronotype could decrease the risk of thyroid cancer in the Finnish population (OR = 0.282, 95%CI: 0.085–0.939). Sleep disorders increased the risk of thyroid cancer in the Finnish population (OR = 2.298, 95%CI: 1.194–4.422). The combined results revealed that sleep duration was correlated with increased risk of thyroid cancer (OR = 5.600, 95%CI: 1.458–21.486).

**Conclusion:**

Decreased sleep duration was associated with increased risk of thyroid cancer, which indicated the importance of adequate sleep for the prevention of thyroid cancer.

**Supplementary Information:**

The online version contains supplementary material available at 10.1186/s12885-024-12376-6.

## Background

Thyroid cancer is the most common endocrine malignancy. According to the Global Cancer Statistics report, the incidence of thyroid cancer ranks ninth among cancers in the world in 2020 [1]. Thyroid cancer is the most prevalent malignancy of the endocrine system, accounting for 3.4% of all annually diagnosed cancers [2]. Although the mortality rate of thyroid cancer is relatively low, the incidence has increased rapidly in recent decades [1, 3]. Identifying modifiable risk factors is important for preventing thyroid cancer and reducing the burden of disease.

In recent years, the relationship between sleep and cancer has been widely concerned [4]. Circadian disturbances could alter the function of the hypothalamic–pituitary–adrenal HPA axis, which in turn regulated thyroid function and may be involved in the development of thyroid tumors [4]. Melatonin played an important role in regulating sleep–wake rhythm, which had antioxidant and inflammatory effects and further inhibited tumor growth [4, 5]. Population-based observational studies have shown that high sleep quality is associated with lower thyroid cancer prevalence [6]. Short sleep duration at night was reported to be associated with a higher incidence of thyroid nodules [7]. However, the association between sleep duration and thyroid cancer was not significant after adjusting for confounding factors in some studies [8]. Traditional epidemiological studies are susceptible to confounding factors and causal inversion, and the causal relationship between sleep characteristics and thyroid cancer risk was still unclear.

Mendelian randomization (MR) employs genetic variation as instrumental variables to investigate the causal relationship between exposures and diseases [9]. MR estimates are less susceptible to bias from potential reverse causality and confounding than traditional observational epidemiological studies [10]. As the genetic code cannot be influenced by environmental factors or preclinical diseases, it is also less susceptible to bias caused by reverse causation [11]. Recently, several MR studies identified a potential causal relationship between sleep traits and the risk of liver cancer, breast cancer and other cancers [12–14]. We suspected that sleep traits might have causal links with the risk of thyroid cancer.

This study intended to explore the causal association between sleep traits and the risk of thyroid cancer based on MR analysis from genome-wide association studies (GWASs) using the FinnGen and UK Biobank databases.

## Methods

### Study design and population

**Fig. 1:**
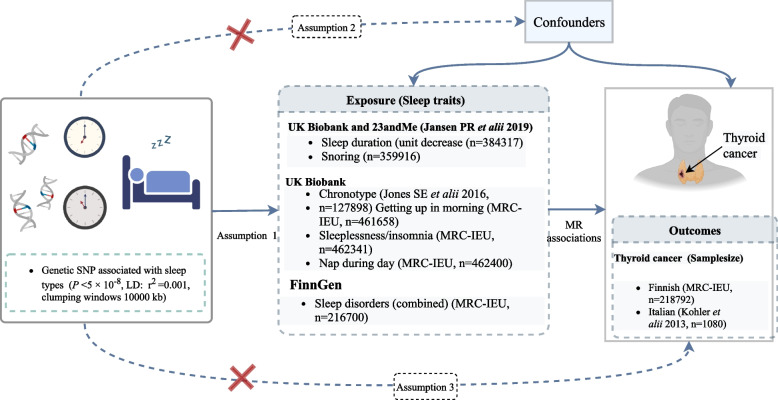
The study design of MR analysis on the causal associations between sleep traits and the risk of thyroid cancer

The study design was shown in Fig. 1. In order to perform our two-sample MR method to explore the causal effects of sleep traits on thyroid cancer, three assumptions were tested. Assumption 1: the selected genetic variants are related to sleep traits; Assumption 2: these genetic variants are not associated with confounders; (3) these genetic variants are related to thyroid cancer only via sleep traits. This study was a two-sample MR analysis with summary single nucleotide polymorphism (SNP)-phenotype association data were obtained from published GWASs data from UK Biobank and FinnGen [IEU OpenGWAS project (mrcieu.ac.uk)] [15–17] of Italian and Finnish population. The summary-level data were de-identified public data and are openly available. Each GWAS involved in this study was ethically approved by the participating centers [18]. The UK Biobank cohort was a prospective population-based study that enrolled over 500,000 adults aged 40–69 years from the general population. The study collected biological samples and a wide range of phenotypic data between April 2006 and December 2010 [19]. The GWAS data of thyroid cancer in Italian population were derived from case–control studies with well-diagnosed cases. FinnGen research project is a public–private partnership combining genotype data from Finnish biobanks and digital health record data from Finnish health registries. FinnGen provides a unique opportunity to study genetic variation in relation to disease trajectories in an isolated population [20]. FinnGen utilizes the extensive longitudinal registry data available on all Finnish, which includes GWAS data on multiple types of diseases, including thyroid cancer. The GWAS data are combined with phenotype data produced from several national health registries, which are relatively reliable. All the GWAS data were publicly available and the populations were of European ancestry. The GWAS data of outcomes in different European populations might be representative of the risk of thyroid cancer in European populations. The data sources of the current study are presented in Table 1. No further informed permission was needed for this study because it only used data that was already available to the public. All studies have received prior approval from the appropriate institutional review boards.

**Table 1:** The data source of genetic instruments for exposures and outcomes

Variables	Phenotype	IEU open GWAS ID	PMID
Exposures	Sleep duration (unit decrease)		30804565
	Snoring		30804565
	Chronotype	ebi-a-GCST003837	27494321
	Sleep disorders (combined)	finn-b-SLEEP	
	Getting up in the morning	ukb-b-2772	
	Sleeplessness/insomnia	ukb-b-3957	
	Nap during day	ukb-b-4616	
Outcomes	Thyroid cancer	ieu-a-1082	23894154
	Malignant neoplasm of thyroid gland	finn-b-C3_THYROID_GLAND	

### Instrument variables

SNPs associated with exposure (sleep duration, snoring, chronotype, sleep disorders, getting up in the morning, sleeplessness/insomnia, and nap during day), and outcome (thyroid cancer) were exhibited in Table 1. SNP associated with chronotype [15], getting up in morning, sleeplessness/insomnia, nap during day, and sleep disorders (combined) were obtained from UK Biobank and FinnGen [IEU OpenGWAS project (mrcieu.ac.uk)]. SNPs associated with sleep duration (1.30-h decrease), and snoring SNPs were sourced from a Meta-GWAS study with data from the UK Biobank (*n* = 449,734) and 23andMe (*n* = 248,098) studies [16]. The instrumental variables were selected based on a series of quality control steps. Firstly, SNPs were genome-wide significant with *P* < 5 × 10^–8^. Secondly, SNPs without linkage disequilibrium (the criteria of clump distance > 10,000 kb and *r* ^2^ < 0.001) were preserved. Thirdly, the relative conservative action was applied to infer positive strand alleles, using allele frequencies for palindromes, and SNPs being palindromic and with minor allele frequency (MAF) < 0.01 were deleted.

### Horizontal pleiotropy analysis

MR-Egger regression was applied to assess the potential pleiotropic effects of the SNPs used as instrument variables. The correlated pleiotropy was contradicted with assumption 2 and uncorrelated pleiotropy was contradicted with assumption 3 [21]. MR analysis should be conducted on the basis of ensuring no horizontal pleiotropy. SNPs directly associated with thyroid cancer, and not through sleep traits were excluded. The MR-PRESSO analysis was utilized to detect the horizontal pleiotropy [22], and the outlier variants with *P* < 0.05 were removed.

### Definitions of the sleep traits

Sleep traits were collected using a standardized touchscreen questionnaire in the UK Biobank [15, 23]. Chronotype is a self-reported measure and individuals were asked to categories themselves as “definitely a ‘morning’ person”, “more a ‘morning’ than ‘evening’ person”, “more an ‘evening’ than a ‘morning’ person”, “definitely an ‘evening’ person” or “do not know” with 127,898 participants. Easiness of getting up in the morning was assessed in the question “on an average day, how easy do you find getting up in the morning?” with one of six possible answers: “not at all easy,” “not very easy,” “fairly easy,” “very easy,” “do not know,” and “prefer not to answer.”, which was evaluated in 461,658 subjects. Nap during day was assessed in 462,400 samples via the question “do you have a nap during day?” with one of the four possible answers: “never/rarely,” “sometimes,” “usually,” and “prefer not to answer.” Sleeplessness/insomnia was assessed in 462,341 subjects through the question “do you have trouble falling asleep at night or do you wake up in the middle of the night?” with one of four possible answers “never/rarely,” “sometimes,” “usually,” and “prefer not to answer.” Sleep duration was assessed in the question “about how many hours do you get in every 24 h? (Please include naps).” The answer could only contain integer values (round hours). Sleep duration was available in 384,317 unrelated individuals of European descent after quality control. The mean and standard deviation (SD) of sleep duration was 7.10 (SD = 1.30) hours per 24 h. In our study, 1-unit sleep duration indicated 1 SD (1.30-h), so we analyzed the causal association of sleep duration (1SD decrease), that was sleep duration (1.30-h decrease) with the risk of thyroid cancer. Snoring was assessed in the question “does your partner or a close relative or friend complain about your snoring?”. Participants could answer with “yes” or “no”. Snoring data were available in 359,916 unrelated individuals of European descent after quality control.

### Statistical analysis

MR analysis was performed to evaluate the causal effects between sleep traits and the risk of thyroid cancer with selected SNPs as instrumental variables. The inverse variance weighted (IVW), the Weighted Median, the Mendelian Randomization robust adjusted profile score (MR-RAPS), the Mendelian randomization pleiotropy residual sum and outlier (MR-PRESSO) methods were employed to evaluate the causal links of sleep traits with thyroid cancer. The IVW method was used as the major analysis method in this study, and the results were combined in UK Biobank and FinnGen studies. The IVW method calculates a weighted average of Wald ratio estimates and is primarily employed for fundamental causal estimates, which would provide the most precise results when all selected SNPs were valid instrument variables [24]. The Weighted median method is effective in preventing the use of invalid tools, and it can also provide consistent estimates of causal effects if 50% of the information is analyzed from genetic variation in invalid instrument variables [25]. MR-RAPS is a robust method to both systematic and idiosyncratic pleiotropy and can give a robust inference for MR analysis with many weak instruments, which is able to correct for pleiotropy using robust adjusted profile scores [26]. The MR-PRESSO analysis detects and attempts to reduce horizontal pleiotropy by removing significant outliers. But the MR-PRESSO outlier test requires that at least 50% of the genetic variants be valid instruments and relies on InSIDE assumptions. Odds ratio (OR) and 95% confidence interval (CI) were calculated. The leave-one-out analysis was employed to assess whether the results were caused by any single SNP associated with sleep traits, and the symmetry in the resulting figure represents no pleiotropy. For testing the results, Cochran’s Q-test was conducted to evaluate the statistical heterogeneity between SNPs in the IVW method, and *P* < 0.05 was set as significantly heterogeneous. The F-statistics and variance explained (R^2^) for each exposure was calculated to assess the instrument variable strength, and F-statistics > 10 were considered to imply adequate instrument strength [27]. F = (N—K—1) / K) × (R^2^ / (1—R^2^). R^2^ = 2 × EAF × (1—EAF) × b × b / SD^2^. (N: Sample size; K: Number of instrument variables; EAF: Effect Allele Frequency; b: beta; SD: Standard difference). Reverse MR analysis was performed to evaluate the causal links between sleep traits and thyroid cancer. All statistical analyses were completed using R 4.1.1 software with the function of “harmonise_data()” and setting harmonise action as 2 in “TwoSampleMR” package.

## Results

### Selection of SNPs and Instrument variables selection and pleiotropy analysis

SNPs strongly associated with sleep traits (*P* < 5 × 10^–8^) were selected. In total, 21 SNPs associated with sleep duration (1.30-h decrease), 42 SNPs associated with snoring, 311 SNPs associated with chronotype, 120 SNPs associated with sleep disorders, 10,132 SNPs associated with getting up in the morning, 2,654 SNPs associated with sleeplessness/insomnia, and 8,636 SNPs associated with napping during day were found in Italian and Finnish population. After omitting SNPs with linkage disequilibrium, and SNPs with palindromic with intermediate allele frequencies, 13 SNPs associated with sleep duration, 15 SNPs associated with snoring, 6 SNPs associated with chronotype, 3 SNPs associated with sleep disorders, 37 SNPs associated with getting up in the morning, 18 SNPs associated with sleeplessness/insomnia, and 49 SNPs associated with napping during day in the Italian population. A total of 18 SNPs associated with sleep duration, 28 SNPs associated with snoring, 10 SNPs associated with chronotype, 3 SNPs associated with sleep disorders, 74 SNPs associated with getting up in the morning, 39 SNPs associated with sleeplessness/insomnia, and 91 SNPs associated with napping during day in the Finnish population were finally included (Table 2).

**Table 2:** The screening procedure of SNPs associated with different sleep traits

Study population	Exposures	SNPs (*P* < 5 × 10^–8^)	SNPs without LD (r^2^, kb:(0.001, 10,000))	SNPs without being palindromic with intermediate allele frequencies	Horizontal pleiotropic test	
					Egger intercept	*P*
Italian	Sleep duration (unit decrease)	21	18	13	-0.0002	0.999
	Snoring	42	31	15	0.1738	0.293
	Chronotype	311	12	6	-0.3168	0.272
	Sleep disorders (combined)	120	3	3	1.2560	0.650
	Getting up in the morning	10,132	76	37	-0.1008	0.219
	Sleeplessness/insomnia	2654	42	18	-0.0079	0.960
	Nap during day	8636	97	49	-0.0456	0.344
Finnish	Sleep duration (unit decrease)	21	18	18	0.0085	0.903
	Snoring	42	31	28	0.0551	0.299
	Chronotype	311	12	10	0.0223	0.826
	Sleep disorders (combined)	120	3	3	0.4777	0.592
	Getting up in the morning	10,132	76	74	-0.0072	0.781
	Sleeplessness/insomnia	2654	42	39	0.0030	0.904
	Nap during day	8636	97	91	0.0297	0.150

*SNP* Single nucleotide polymorphism, *LD* Linkage disequilibrium

### MR analysis for causal links of sleep traits with the risk of thyroid cancer

**Fig. 2:**
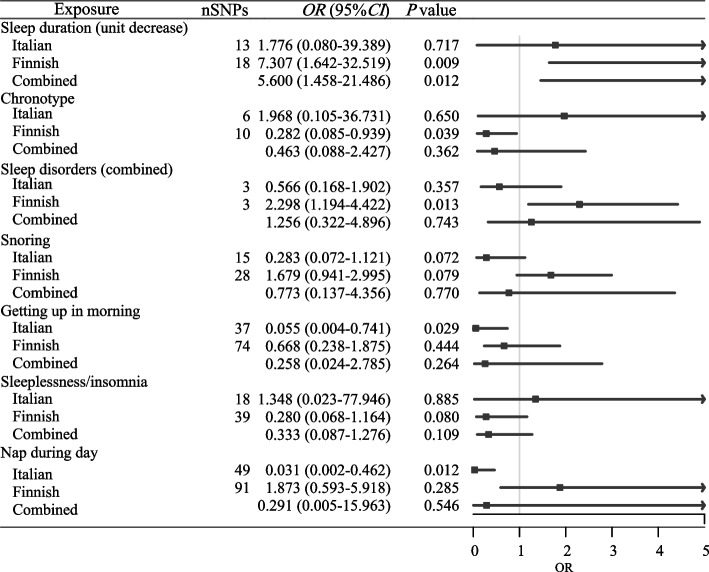
The results of IVW showing the causal associations between sleep traits and the risk of thyroid cancer

**Fig. 3:**
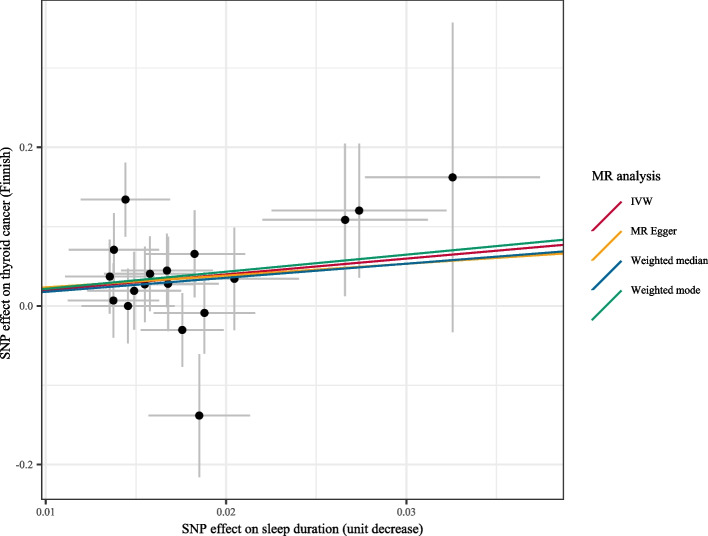
The scatter plot of the results from weighted median/MR-RAPS/MR-PRESSO on the association between sleep duration and thyroid cancer in Finnish population

As shown in Table 3, getting up in the morning (OR = 0.055, 95%CI: 0.004–0.741) and napping during day (OR = 0.031, 95%CI: 0.002–0.462) were associated with decreased risk of thyroid cancer in the Italian population. Sleep duration reduction could increase the risk of thyroid cancer in the Finnish population (OR = 7.307, 95%CI: 1.642–32.519). Chronotype could decrease the risk of thyroid cancer in the Finnish population (OR = 0.282, 95%CI: 0.085–0.939). Sleep disorders increased the risk of thyroid cancer in the Finnish population (OR = 2.298, 95%CI: 1.194–4.422). The combined results revealed that a 1.30-h decrease of sleep duration was correlated with 5.600-fold of increase risk of thyroid cancer (OR = 5.600, 95%CI: 1.458–21.486) (Fig. 2). The results of causal links of sleep traits with the risk of thyroid cancer using weighted median/MR-RAPS/MR-PRESSO were exhibited in Supplementary Table 1. The scatter plot of the results from weighted median/MR-RAPS/MR-PRESSO on the association between sleep duration decrease and thyroid cancer in Finnish population was presented in Fig. 3.

**Table 3:** The results of MR analysis on the association between different sleep traits and thyroid cancer

Outcome (Consortium)	Exposure	SNPs (n)	IVW	
			OR (95%CI)	*P*
Thyroid cancer (Italian)	Sleep duration (unit decrease)	13	1.776 (0.080–39.389)	0.717
	Chronotype	6	1.968 (0.105–36.731)	0.650
	Sleep disorders (combined)	3	0.566 (0.168–1.902)	0.357
	Snoring	15	0.283 (0.072–1.121)	0.072
	Getting up in the morning	37	0.055 (0.004–0.741)	0.029
	Sleeplessness/insomnia	18	1.348 (0.023–77.946)	0.885
	Nap during day	49	0.031 (0.002–0.462)	0.012
Thyroid cancer (Finnish)	Sleep duration (unit decrease)	18	7.307 (1.642–32.519)	0.009
	Chronotype	10	0.282 (0.085–0.939)	0.039
	Sleep disorders (combined)	3	2.298 (1.194–4.422)	0.013
	Snoring	28	1.679 (0.941–2.995)	0.079
	Getting up in the morning	74	0.668 (0.238–1.875)	0.444
	Sleeplessness/insomnia	39	0.280 (0.068–1.164)	0.080
	Nap during day	91	1.873 (0.593–5.918)	0.285
Combined results	Sleep duration (unit decrease)	-	5.600 (1.458–21.486)	0.012
	Chronotype	-	0.463 (0.088–2.427)	0.362
	Sleep disorders (combined)	-	1.256 (0.322–4.896)	0.743
	Snoring	-	0.773 (0.137–4.356)	0.770
	Getting up in the morning	-	0.258 (0.024–2.785)	0.264
	Sleeplessness/insomnia	-	0.333 (0.087–1.276)	0.109
	Nap during day	-	0.291 (0.005–15.963)	0.546

*SNP* Single nucleotide polymorphism, *OR* Odds ratio, *CI* Confidence interval, *MR* Mendelian randomization, *IVW* Inverse variance weighted

The MR-Egger analysis showed that there was no pleiotropic bias in the remaining SNPs. The results of instrumental variable strength and heterogeneity test were presented in Table 4. There were strong associations of sleep duration associated SNPs (F = 37), snoring associated SNPs (F = 44), chronotype associated SNPs (F = 36), sleep disorders (F = 47), getting up in the morning associated SNPs (F = 44), sleeplessness/insomnia associated SNPs (F = 40), and nap during day (F = 97), and thyroid cancer in the Italian population. There were strong associations of sleep duration associated SNPs (F = 37), snoring associated SNPs (F = 47), chronotype associated SNPs (F = 37), sleep disorders (F = 47), getting up in the morning associated SNPs (F = 43), sleeplessness/insomnia associated SNPs (F = 45), and nap during day (F = 46), and thyroid cancer in the Finnish population.

**Table 4:** Test for strength and heterogeneity of the results of MR analysis

Outcomes	Exposures	Horizontal pleiotropic test		Heterogeneity test				Strength		MR-PRSSO		
		Egger intercept	*P*	MR Egger Q	*P*	IVW Q	*P*	*F*-value	*R* ^*2*^(%)	Global Test	*P*	Outlier SNP
Italian	Sleep duration (unit decrease)	-0.0002	0.999	27.30	0.004	27.30	0.007	37	10.61	32.88	0.005	rs12607679
	Snoring	0.1738	0.293	20.72	0.079	22.64	0.066	44	13.11	25.36	0.075	-
	Chronotype	-0.3168	0.272	2.84	0.584	4.46	0.485	36	1.10	6.37	0.505	-
	Getting up in morning	-0.1008	0.219	40.72	0.233	42.54	0.210	44	3.12	44.82	0.204	-
	Sleeplessness / insomnia	-0.0079	0.960	23.63	0.098	23.64	0.130	40	5.67	26.48	0.132	-
	Nap during day	-0.0456	0.344	48.41	0.416	49.35	0.419	48	0.06	52.07	0.416	-
	Sleep disorders (combined)	1.2560	0.650	4.79	0.029	6.58	0.037	47	1.89	-	-	-
Finnish	Sleep duration (unit decrease)	0.0085	0.903	16.10	0.446	16.11	0.516	37	15.62	17.95	0.535	-
	Snoring	0.0551	0.299	32.51	0.177	33.91	0.169	47	21.20	37.18	0.145	-
	Chronotype	0.0223	0.826	19.01	0.015	19.13	0.024	37	8.10	22.87	0.043	rs2050122
	Getting up in morning	-0.0072	0.781	64.15	0.734	64.23	0.759	43	8.08	66.00	0.762	-
	Sleeplessness / insomnia	0.0030	0.904	31.08	0.742	31.09	0.779	45	0.50	32.75	0.793	-
	Nap during day	0.0297	0.150	71.04	0.919	73.15	0.902	46	8.83	74.64	0.891	-
	Sleep disorders (combined)	0.4777	0.592	1.92	0.165	3.00	0.223	47	1.89	-	-	-

*MR* Mendelian randomization, *IVW* Inverse variance weighted, *SNP* Single nucleotide polymorphism

### Sensitivity analysis

The results of leave-one-out analysis depicted that getting up in the morning (Supplementary Fig. 1), and nap during day (Supplementary Fig. 2) had a strong causal association with the risk of thyroid cancer in the Italian population.

**Fig. 4:**
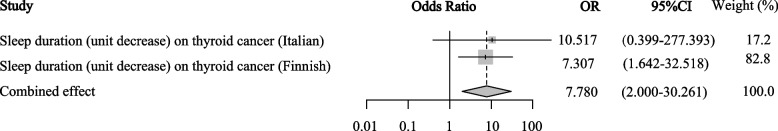
MR results of the causal association of sleep duration (1.30-h decrease) and the risk of thyroid cancer was statistical in Finnish population excluding the outlier SNP

In the Finnish population, the causal associations of sleep duration (Supplementary Fig. 3), and sleep disorders (Supplementary Fig. 4) with the risk of thyroid cancer were robust in Finnish population. As for the causal association between chronotype and the risk of thyroid cancer, rs10157197, rs1075265, rs113240734, rs12140153, rs2050122, rs2653344, rs4821940, rs516134, rs77641763 and rs9961653 were gradually removed by leave-one-out method, and the results suggested that the causal association between chronotype and the risk of thyroid cancer was not statistically significant. rs2050122 was an outlier SNP according to MR-PRESSO results (Table 4). The MR results excluded the outlier SNP were exhibited in Fig. 4, which revealed that the causal association of sleep duration decrease and the risk of thyroid cancer was statistical in Finnish population.

In the Italian population, Cochran’s Q of the MR-Egger and IVW methods showed heterogeneity in the association between sleep duration decrease and the risk of thyroid cancer. rs12607679 was an outlier SNP detected by MR-PRESSO. After removing this SNP, the difference was not statistically significant, and the result was similar with the IVW result (Table 4).

### Reverse MR analysis

**Fig. 5:**
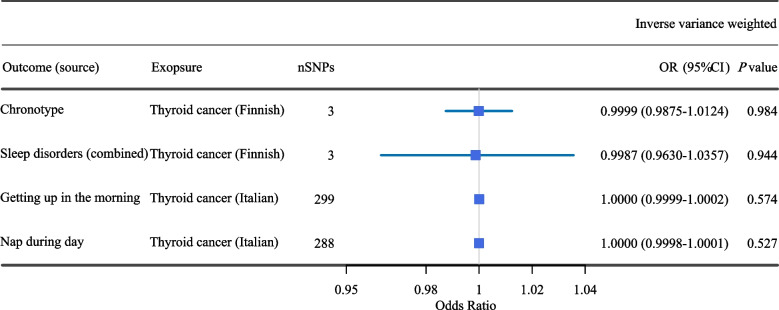
The forest plot presenting the results of reverse MR analysis on the causal associations between thyroid cancer (exposure) and the risk of sleep traits (outcomes)

We performed an additional reverse MR analysis to explore reverse causality. Significant reverse MR analysis indicates reverse causality from thyroid cancer (as exposure) to sleep traits (as outcome). The reverse MR analysis procedure was the same as the above MR analysis. The results of the reverse MR analysis showed no evidence of causal effect from thyroid cancer to sleep traits (Fig. 5).

## Discussion

The present study explored the causal association between sleep traits and the risk of thyroid cancer using a MR analysis. The results revealed that getting up in the morning was associated with decreased risk of thyroid cancer genetically in the Italian population, while decreased sleep duration and sleep disorders were causally associated with increased risk of thyroid cancer in the Finnish population. The findings might provide evidence for the prevention of thyroid cancer and screening of people who were at high-risk of thyroid cancer.

The importance of sleep cannot be overstated in maintaining human health, as it significantly impacts a wide array of crucial physiological functions [28]. The regulation of sleep is a complex process that is tightly controlled by physiological mechanisms [29]. In the current study, we found that chronotype and getting up in the morning had causal association with decreased risk of thyroid cancer. Chronotype and getting up in the morning are closely related to circadian rhythms, which have been identified as risk factors for cancers such as breast cancer and epithelial ovarian cancer [30]. The production of almost all hormones follows a cyclical rhythm within 24-h intervals, and the regulation of this rhythm is influenced by sleep to varying degrees [31]. Chronic sleep dept disrupts rhythmic thyroid-stimulating hormone (TSH) secretion [32], and elevated serum TSH level was reported to be associated with the incidence of human thyroid cancer [33]. Although the hypothalamic-pituitary-thyroid axis is regulated by the circadian clock through the suprachiasmatic nucleus pacemaker, some patients with hypothyroidism and hyperthyroidism experience disruptions in their daily profiles of TSH secretion [5]. Disruption of the circadian timing system caused by circadian misalignment could be responsible of several types of cancers [34]. Dysregulation of the clock system can influence cancer susceptibility by regulating DNA damage and repair mechanisms, as well as apoptosis, and there were findings suggesting that clock genes are associated with follicular and papillary thyroid carcinomas and parathyroid adenomas [35]. In this study, the results showed some difference between Italian and Finish populations, and the variability in results might due to the different outcome diagnosis and definitions. In the Italian population, thyroid cancer was histological proved through the Cisanello Hospital in Pisa [17]. In the Finnish population, the cases of thyroid cancer were diagnosed by the code of C73 of ICD-10 (International Statistical Classification of Diseases and Related Health Problems 10th Revision) or 193 of ICD-9/8. The control for thyroid cancer were individuals that are not cases (https://risteys.finregistry.fi/endpoints/C3_THYROID_GLAND). Also, the population stratification might have bias [36], indicating the limitations of the generalizability of the findings. Notably, the quality and duration of sleep have a significant impact on TSH levels, and there is also a 24-h circadian rhythm observed in free triiodothyronine (FT3) that parallels TSH [37]. A NIH-AARP Diet and Health Study indicated that sleep duration < 5 h was correlated with increased risk of thyroid cancer compared with sleep duration of 7–8 h [8]. This might provide support to the findings in our study, which depicted that decreased sleep duration was genetically associated with increased risk of thyroid cancer. The linear association between sleep duration and risk of thyroid cancer did not analyzed in previous studies, and in our MR analysis, the linear association between sleep duration and risk of thyroid cancer could not be analyzed.

The current study evaluated the causal links between sleep traits and the risk of thyroid cancer via MR, and reverse MR analysis, which might provide evidence for the prevention and treatment of thyroid cancer. Sleep is important for common people, and adequate sleep and keep appropriate circadian rhythms might be essential for thyroid cancer prevention. Several limitations were found in our study. Firstly, all data were individuals of European descent, and the results might not suitable for other ethnic groups. Secondly, due to the limited data of some sleep traits, the results of reverse MR analysis such as sleep duration were not analyzed. Thirdly, the confidence intervals were wide, this might be related to the small sample size of the selected outcome data. In Italian population, 701 patients with thyroid cancer and 499 controls while in the Finnish population, 989 thyroid cancer cases and 217,803 controls were included. Compared with the exposure in GWAS data, the sample size of the outcome data in both regions was relatively small and the number of cases was relatively small, which may affect the stability of the estimates. The generalization of the results to the target population should be done with caution. In the future, it is necessary to further confirm the causal relationship between sleep duration and thyroid cancer based on larger sample size and more instrumental variables.

## Conclusions

The causal associations between sleep traits and the risk of thyroid cancer were analyzed using a MR analysis, which delineated that reduced sleep duration was associated with elevated risk of thyroid cancer. The results might provide evidence for the prevention and management of thyroid cancer via controlling sleep.

### Supplementary Information

Supplementary Material 1.

Supplementary Material 2.

Supplementary Material 3.

Supplementary Material 4.

Supplementary Material 5.

## Data Availability

The datasets generated and/or analyzed during the current study are available in the GWAS, https://gwas.mrcieu.ac.uk/.

## Abbreviations

MR: Mendelian randomization
GWASs: Genome-wide association studies
SNP: Single nucleotide polymorphism
IVW: Inverse variance weighted
MR-RAPS: Mendelian Randomization robust adjusted profile score
OR: Odds ratio
CI: Confidence interval
TSH: Thyroid-stimulating hormone
FT3: Free triiodothyronine
