# Supplementary material for: Causal association of sleep traits with the risk of thyroid cancer: A mendelian randomization study
Source: BMC Cancer. 2024 May 17;24:605. doi: 10.1186/s12885-024-12376-6 (PMC11102272; doi:10.1186/s12885-024-12376-6)
Supplement: Supplementary file 1 — Supplementary Material 1. [file 12885_2024_12376_MOESM1_ESM.docx]

Supplementary Table 1 The result of causal links of sleep traits with the risk of thyroid cancer using weighted median\MR-RAPS\MR-PRESSO

| Outcome and exposure | SNPs (n) | Weighted median |  | MR-RAPS |  | MR-PRESSO |  |
| --- | --- | --- | --- | --- | --- | --- | --- |
|  |  | *OR* (95%*CI*) | *P* | *OR* (95%*CI*) | *P* | *OR* (95%*CI*) | *P* |
| Thyroid cancer (Italy) |  |  |  |  |  |  |  |
| Chronotype | 6 | 2.91 (0.08-112.56) | 0.566 | 0.04 (0.00-0.54) | 0.016 | 1.34 (0.40-4.46) | 0.651 |
| Sleep disorders (combined) | 3 | 0.62 (0.11-3.46) | 0.589 | - |  | - | - |
| Sleep duration (unit decrease) | 13 | 18.39 (0.19-1814.91) | 0.214 | 1.84 (0.08-42.34) | 0.703 | 1.78 (0.02-190.44) | 0.814 |
| Snoring | 15 | 0.19 (0.03-1.32) | 0.093 | 1.69 (0.97-2.94) | 0.065 | 0.28 (0.05-1.63) | 0.179 |
| Getting up in the morning | 37 | 0.08 (0.00-4.41) | 0.221 | 1.21 (0.30-4.83) | 0.788 | 0.06 (0.00-0.93) | 0.052 |
| Sleeplessness/insomnia | 18 | 0.89 (0.00-326.29) | 0.970 | 3.86 (0.07-198.93) | 0.502 | 1.35 (0.01-161.27) | 0.904 |
| Nap during day | 49 | 0.09 (0.00-5.57) | 0.256 | 0.54 (0.16-1.85) | 0.330 | 0.03 (0.00-0.48) | 0.016 |
| Thyroid cancer (Finnish) |  |  |  |  |  |  |  |
| Chronotype | 10 | 2.33 (0.47-11.44) | 0.299 | 0.61 (0.36-1.00) | 0.053 | 0.33 (0.07-1.67) | 0.210 |
| Sleep disorders (combined) | 3 | 1.82 (0.82-4.03) | 0.142 | - |  | - | - |
| Sleep duration (unit decrease) | 18 | 5.90 (0.78-44.60) | 0.086 | 7.61 (1.62-35.78) | 0.010 | 7.31 (1.71-31.26) | 0.016 |
| Snoring | 28 | 2.12 (0.88-5.10) | 0.095 | 1.69 (0.97-2.94) | 0.065 | 1.68 (0.88-3.21) | 0.129 |
| Getting up in the morning | 74 | 0.79 (0.18-3.52) | 0.759 | 0.71 (0.25-2.02) | 0.525 | 0.67 (0.25-1.76) | 0.417 |
| Sleeplessness/insomnia | 39 | 0.56 (0.07-4.44) | 0.580 | 0.35 (0.09-1.43) | 0.143 | 0.28 (0.08-1.02) | 0.060 |
| Nap during day | 91 | 1.20 (0.23-6.18) | 0.831 | 2.34 (1.18-4.63) | 0.015 | 1.88 (0.66-5.28) | 0.239 |

SNP, single nucleotide polymorphism; *OR*, odds ratio; *CI*, confidence interval; IVW, inverse variance weighted; MR, mendelian randomization; MR-RAPS, the Robust Adjusted Profile Score of MR; MR-PRESSO, MR Pleiotropy RESidual Sum and Outlier tests.
