# Supplementary figures and images for: Causal association of sleep traits with the risk of thyroid cancer: A mendelian randomization study
Source: BMC Cancer. 2024 May 17;24:605. doi: 10.1186/s12885-024-12376-6 (PMC11102272; doi:10.1186/s12885-024-12376-6)

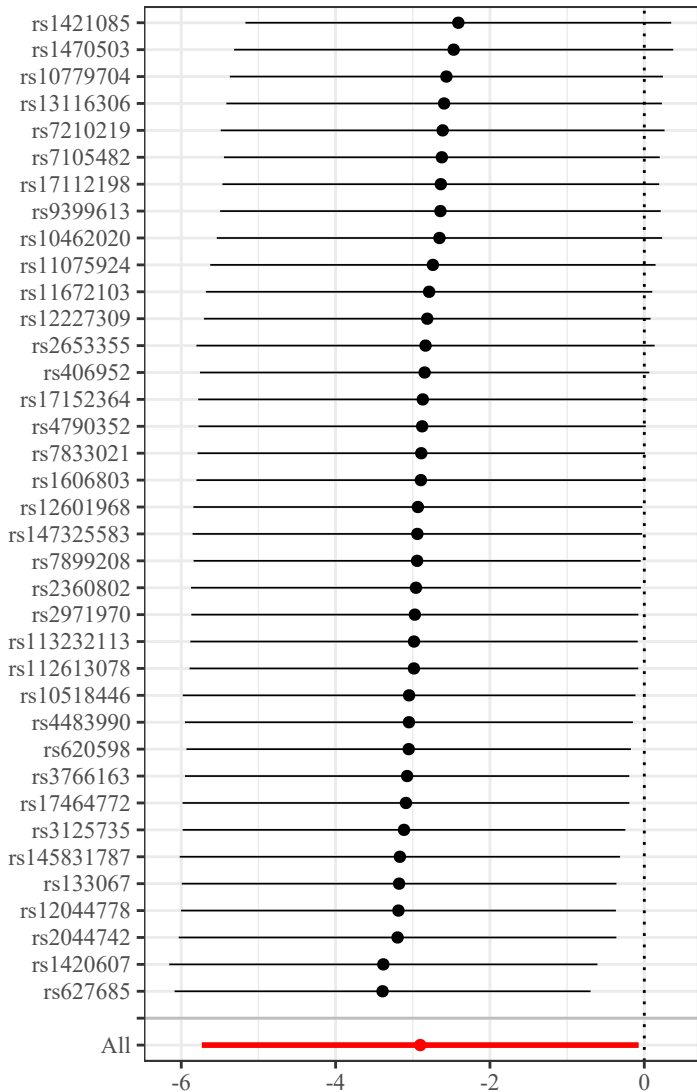

MR leave-one-out sensitivity analysis for getting up in the morning on thyroid cancer (Italy)

Supplement: Supplementary file 2 — Supplementary Material 2. [file 12885_2024_12376_MOESM2_ESM.pdf]

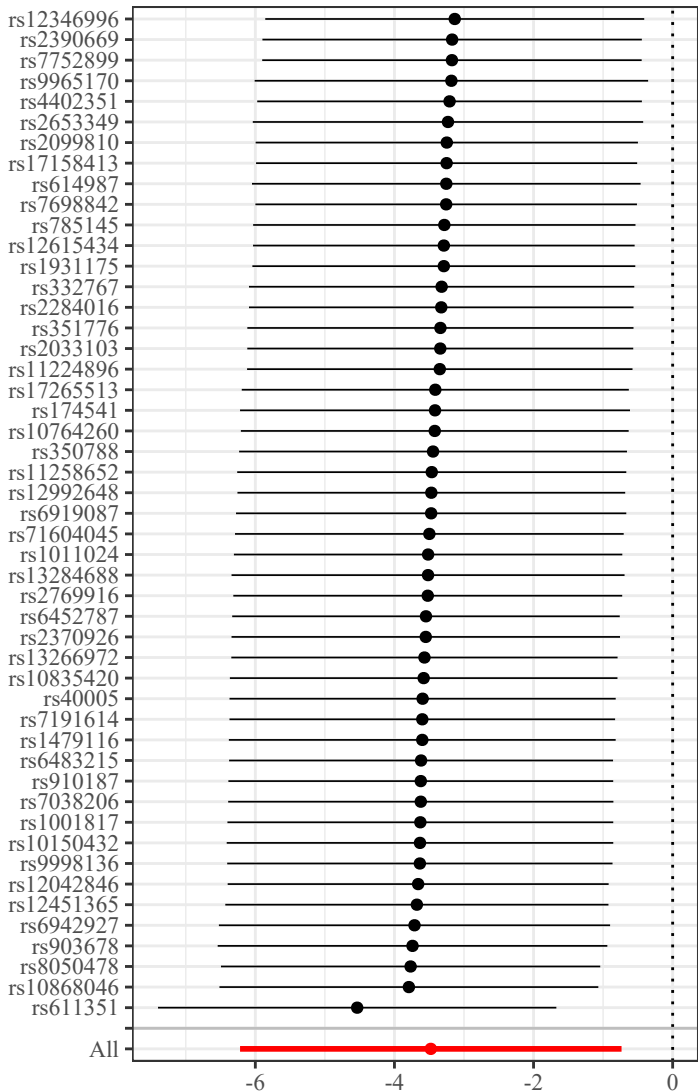

MR leave-one-out sensitivity analysis for  
nap during day on thyroid cancer (Italy)

Supplement: Supplementary file 3 — Supplementary Material 3. [file 12885_2024_12376_MOESM3_ESM.pdf]

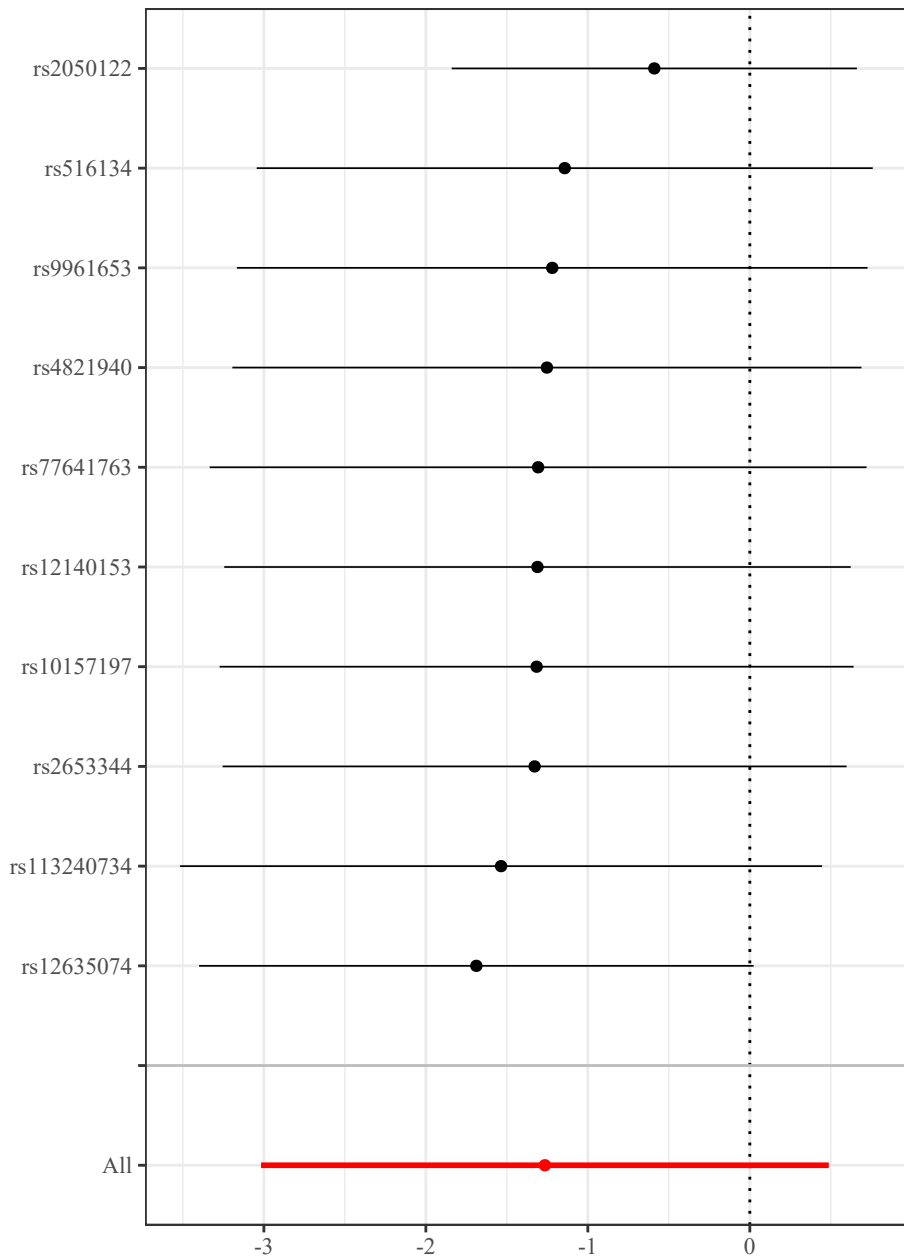

MR leave-one-out sensitivity analysis for  
chronotype on thyroid cancer (Finnish)

Supplement: Supplementary file 5 — Supplementary Material 5. [file 12885_2024_12376_MOESM5_ESM.pdf]
