# Supplementary material for: Causal association of sleep traits with the risk of thyroid cancer: A mendelian randomization study
Source: BMC Cancer. 2024 May 17;24:605. doi: 10.1186/s12885-024-12376-6 (PMC11102272; doi:10.1186/s12885-024-12376-6)
Supplement: Supplementary file 4 — Supplementary Material 4. [file 12885_2024_12376_MOESM4_ESM.pdf]

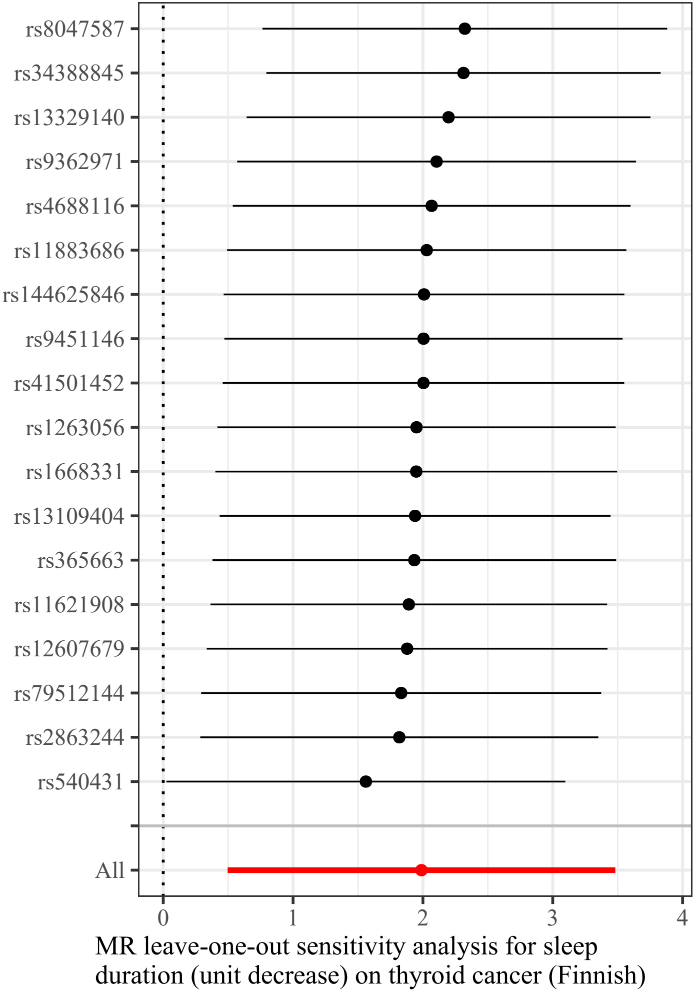
rs8047587
rs34388845
rs13329140
rs9362971
rs4688116
rs11883686
rs144625846
rs9451146
rs41501452
rs1263056
rs1668331
rs13109404
rs365663
rs11621908
rs12607679
rs79512144
rs2863244
rs540431
All
0
1
2
3
4
MR leave-one-out sensitivity analysis for sleep duration (unit decrease) on thyroid cancer (Finnish)
